# Real-time Associations between Digital Indicators and Daily Pain and Depressive Symptoms among Korean Older Adults

**DOI:** 10.1093/geroni/igaf122.4307

**Published:** 2025-12-31

**Authors:** Sunmi Song, Seo-Yeon Hwang, Hae-Young Kim, Junesun Kim

**Affiliations:** Korea University, Seoul, Republic of Korea; Korea University, Seoul, Republic of Korea; Korea University, Seoul, Republic of Korea; Korea University, Seoul, Republic of Korea

## Abstract

Despite the importance of personalized digital healthcare services for addressing pain and emotional health in older adults, the development of digital healthcare interventions has been limited by a lack of evidence on real-time predictors of pain and emotional health among older adults. This study examined whether digital sensing data on heart rate variability, sleep quality, and physical activity could predict same-day or next-day pain and depressive symptoms among socially vulnerable older adults. As part of a larger project evaluating the efficacy of a digital healthcare platform integrated with a public community-based care service, older adult care recipients (n = 35; Mean age = 78.03, SD = 4.10; 80% women) and their community caregivers (n = 16) participated in a 6-week trial. Depressive symptoms were assessed daily using the 9-item Patient Health Questionnaire via scripted chatbot interviews, while pain was measured using a Visual Analogue Scale. Digital biomarkers—including heart rate variability, sleep, and physical activity—were collected via a continuously worn wearable sensor (Fitbit). Multilevel modeling revealed that within-person fluctuations in heart rate variability, mean of normal-to-normal intervals (Mean NNI; p = .016), and moderate physical activity (p = .001) were associated with same-day pain. Changes in sleep duration (p = .031) and efficiency (p = .053) were linked to same-day depressive symptoms. Lagged models further showed that Mean NNI (p = .031) and sleep fragmentation (p = .027) predicted next-day pain. These findings highlight the potential of real-time wearable data to inform personalized pain and mental health management for older adults.

